# The use of the Scar Cosmesis Assessment and rating scale to evaluate the cosmetic outcomes of totally thoracoscopic cardiac surgery

**DOI:** 10.1186/s13019-020-01294-w

**Published:** 2020-09-11

**Authors:** Ling-chen Huang, Dao-zhong Chen, Liang-wan Chen, Qi-chen Xu, Zi-he Zheng, Xiao-fu Dai

**Affiliations:** Department of Cardiovascular Surgery, Union Hospital, Fujian Medical University, Fuzhou, 350001 People’s Republic of China

**Keywords:** Median Sternotomy, Totally thoracoscopic, Cardiac surgery, Cosmetic outcomes, Scar assessment

## Abstract

**Background:**

Conventional median sternotomy is widely used in cardiac surgery, while thoracoscopic cardiac surgery, which is considered to have aesthetic advantages, is being performed increasingly more often in China because patients’ requests for minimally invasive procedures yielding aesthetically pleasing results have significantly increased. Few studies have been conducted to assess surgical scars after cardiac surgery. Compared to the median sternotomy approach, multiple-incision totally thoracoscopic cardiac surgery requires smaller but numerous and scattered incisions. In addition to two working ports on the upper and lower margins of the right breast, an inguinal incision and an axillary incision are made. Therefore, does totally thoracoscopic cardiac surgery truly have aesthetic advantages? This study has the following objectives: (a) to compare median sternotomy cardiac surgery and total thoracoscopic cardiac surgery in terms of the long-term cosmetic outcomes of post-operative scars and (b) to evaluate the effectiveness of the Scar Cosmesis Assessment and Rating scale in combination with the numeric rating scale in the assessment of surgical scars after cardiac surgery.

**Methods:**

Consecutive patients who visited our institution from January 2019 to May 2019 for cardiac surgery via median sternotomy or the totally thoracoscopic approach and followed up for at least one year were included. Inter-rater reliability, internal consistency and convergent validity were evaluated for the Scar Cosmesis Assessment and Rating scale and the numeric rating scale. Clinical characteristics and the scores of the two scales were compared between the two groups using Student’s t test or the Mann-Whitney U test.

**Results:**

Thirty-one patients underwent cardiac surgery via the totally thoracoscopic approach, and 42 patients underwent cardiac surgery via the median sternotomy approach. No significant differences were found in the demographic or clinical data between the two groups. The validity and reliability of the two scales were satisfactory. For the Scar Cosmesis Assessment and Rating scale, the median sternotomy group scored statistically significantly higher than did the totally thoracoscopic group on the “overall impression” and “patient question” subscales (*P* < 0.05). The overall scores of the Scar Cosmesis Assessment and Rating scale and numeric rating scale were statistically significantly different (*P* < 0.05).

**Conclusions:**

The Scar Cosmesis Assessment and Rating scale in combination with the numeric rating scale is an effective tool for the assessment of scar aesthetics after cardiac surgery. Surgical scars of totally thoracoscopic cardiac surgery can yield desirable cosmetic outcomes in Chinese individuals, especially in susceptible individuals with a high risk of keloid and hypertrophic scars. Patients with appropriate indications can undergo cardiac surgery with the totally thoracoscopic approach and exhibit a satisfactory scar appearance.

## Introduction

Median sternotomy is the most common surgical method used to access the heart and is widely used worldwide [1]**.** Since Cosgrove [2] and Carpentier [3] performed minimally invasive cardiac surgery,

with the advancement of technology, and the accompanying improvements in extracorporeal circulation and surgical techniques, minimally invasive totally thoracoscopic cardiac surgery is being performed increasingly more often in clinical practice. Many studies have focused on the clinical effect and postoperative complications of this surgery [4], but relatively little research has been conducted on the cosmetic outcomes, especially in the Chinese population. In clinical practice, it is well known that thoracoscopic cardiac surgery has smaller but distributed incision. Three thoracoscopic ports were required to be placed in the chest wall. Whether the scars are more aesthetically pleasing than those of the median sternotomy has not been studied, so this study is of clinical significance.

Postoperative scar formation is inevitable after surgery and can affect the physical and mental health of the patient. There are three distinct phases in the classic model of wound healing, inflammation, proliferation and the remodelling phase, and it takes at least one year for the scar to mature [5], so scars need to be assess over a long follow-up period. Previously, there was no ideal valid and reliable scar scale that effectively assessed postoperative scarring in terms of aesthetics and function. However, the Scar Cosmesis Assessment and Rating (SCAR) Scale, which was proposed in 2016, was initially developed to be used for the assessment of linear incisions after surgery. There is an advantage for this scale; it can be assessed by photographs. The SCAR scale includes six clinician-related questions scored by observers and two simple questions regarding symptoms (itch and pain) with yes/no answer options that are answered by the patient [6]. This scale incorporates objective measures and patient-reported symptoms, its convergent validity, interrater reliability and intra-rater reliability have been tested, and it has shown outstanding integrative results in terms of feasibility, validity and reliability in postsurgical scar-related outcome measures [7]. The reliability of the SCAR scale in assessing scars has been validated; however, its application in totally thoracoscopic and median sternotomy approach cardiac surgery has not been assessed.

In addition to scar features, the cosmetic appearance from a patient’s perspective is integral and should be included in comprehensive scar assessments. We used the numerical rating scale (NRS), with scores ranging from 0 to 10, to assess the cosmetic appearance of the scar from the patients’ perspective. The NRS is a widely used tool for evaluating clinical outcomes and is usually used for the assessment of pain intensity but is also used for the assessment of cosmetic results [8–10].

This study has the following objectives: (a) to compare median sternotomy cardiac surgery and total thoracoscopic cardiac surgery in terms of the long-term cosmetic appearance of post-operative scars and (b) to evaluate the effectiveness of the SCAR scale in combination with the NRS in the assessment of surgical scars after cardiac surgery.

## Materials and methods

### Patients

The clinical data of the patients who visited our department from January 2019 to May 2019 to undergo primary cardiac surgery using cardiopulmonary bypass with median sternotomy or the totally thoracoscopic approach and were followed up for at least one year were collected. All the participants had heart disease, including mitral valve disease, tricuspid valve disease, atrial septal defects, and comorbid atrial fibrillation, which can be treated with thoracoscopic guidance, and all the patients had been given alternative treatment options to surgery during the pre-operative interviews. The inclusion criteria were as follows: (1) primary cardiac surgery could be performed thoracoscopically; (2) no prior right thoracic surgery; (3) complete a whole-course follow-up. The exclusion criteria were as follows: (1) the inability to undergo a routine examination; (2) hearing disorders; (3) significant peripheral vascular disease; (4) severe cardiac insufficiency; (5) severe pectus excavatum and kyphoscoliosis; and (6) the need for additional aortic valve surgery and/or coronary artery bypass grafting. Severe adverse events were defined according to the guidelines published by Akins [11]. This study was approved and monitored by the ethics committee of the Fujian Union Hospital. All participants included in this study signed a written informed consent form.

### Surgical technique

**Fig. 1:**
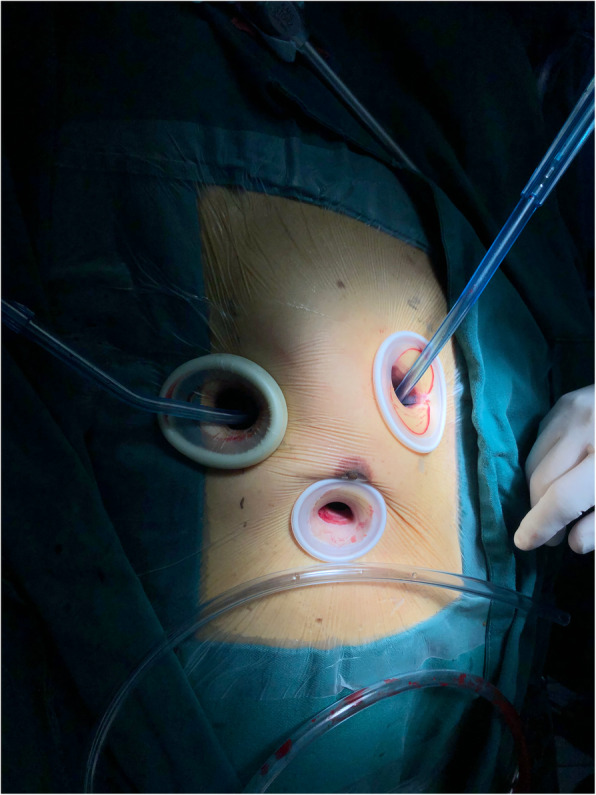
Totally thoracoscopic incision with soft tissue retractor in place

All the surgeries were performed by the same team of experienced surgeons who had already completed the learning curve. The incision for thoracoscopic surgery was made via right endoscopic minithoracotomy. The primary incision measured approximately 2–4 cm and was located on the right midaxillary line in the fourth intercostal space, according to the position of the hilum of the lung on the chest film. We used soft tissue retractors to increase exposure without rib spreading and to protect the wound incisions (Fig. 1). Two additional thoracic working ports were installed in the secondary and fifth intercostal spaces for manipulation and insertion of the prostheses. The second thoracic working port, measuring approximately 2–4 cm, was located in the second intercostal space on the right side of the sternum and was used for the insertion of surgical instruments, such as the tissue forceps or valve prostheses, using the left hand (for a right-handed operator). The third working port, measuring approximately 2 cm, was handled by the right hand of the operator and was usually located in the fourth or fifth intercostal space on a midclavicular line (located on the lower margin of the breast as far as possible without affecting the operation). A longitudinal incision of approximately 3–4 cm was made vertical to the inguinal crease to access the femoral vessels. The sternotomy incision was made carefully on the midline of the sternum.

**Fig. 2:**
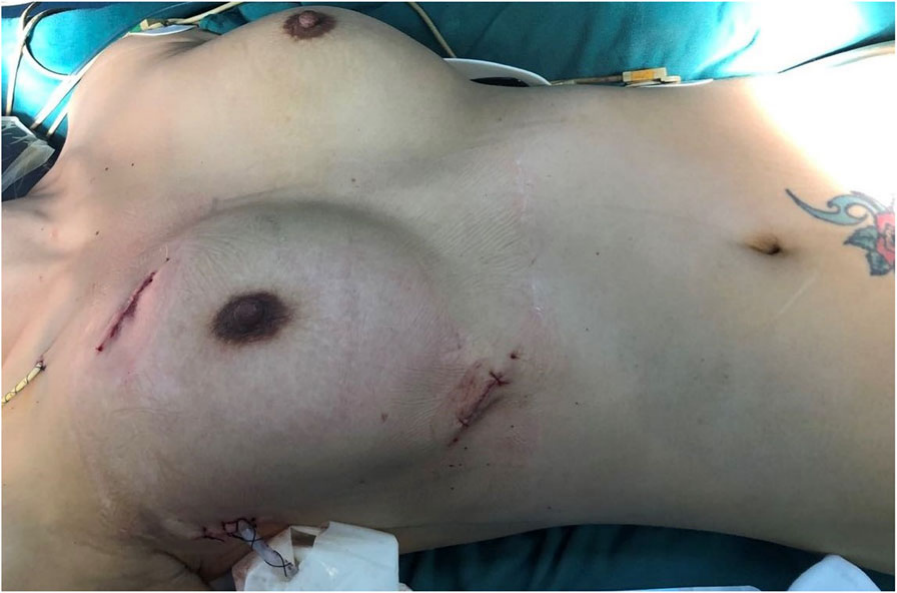
Sutured incision after the totally thoracoscopic cardiac surgery

We used non-absorbable sutures to close the subcutaneous tissue for all the surgical incisions. The continuous suture technique with 3–0 prolene was used in the mid-level dermis. A U-shaped suture was used to close the wound for the drainage tube (Fig. 2).

### Scar assessment

The SCAR scale consists of two parts: clinician-related questions and patient-related questions. There are six clinical items, scored by the observer, and two simple yes/no patient-related questions about pain and itch that are answered by the patient. The scores can be determined through direct observation and evaluation or through the use of high-quality photographic images [12]. The patient-related questions can be answered by the patient verbally or by paper. In totally thoracoscopic cardiac surgery, a total of four incisions are made, and the highest score is selected as the final score (Table 1).

**Table 1:** The Scar Cosmesis Assessment and Rating (SCAR) scale

Parameter	Descriptor	Score
Clinician questions		
Scar spread	None/near invisible	0
	Pencil-thin line	1
	Mild spread, noticeable on close inspection	2
	Moderate spread, obvious scarring	3
	Severe spread	4
Erythema	None	0
	Light pink, some telangiectasias may be present	1
	Red, many telangiectasias may be present	2
	Deep red or purple	3
Dyspigmentation	Absent	0
	Present	1
Suture marks	Absent	0
	Present	1
Hypertrophy/atrophy	None	0
	Mild: palpable, barely visible hypertrophy or atrophy	1
	Moderate: clearly visible hypertrophy or atrophy	2
	Severe: marked hypertrophy or atrophy or keloid formation	3
Overall impression	Desirable scar	0
	Undesirable scar	1
Patient questions		
Itch	No	0
	Yes	1
Pain	No	0
	Yes	1

**Fig. 3:**
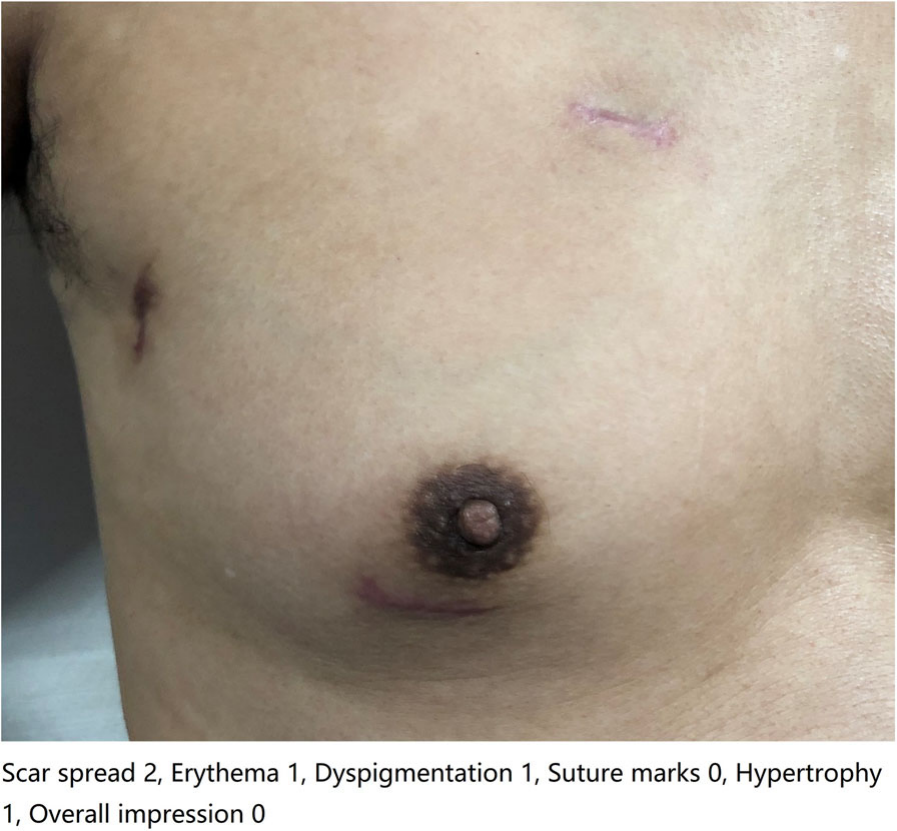
The application of the SCAR scale in study cases of the totally thoracoscopic group

All the scars were assessed and photographed under standard conditions and then scored based on the SCAR scale and the NRS. Each scar was rated by two independent observers who had been trained to use the SCAR scale. The observers were experienced residents who were experts in the patients’ follow-up care. The scores given by this pair of observers were documented for the reliability test. The two observers then discussed and assessed each scar and gave a final score. If the two observers disagreed with this score, a third observer (experienced surgeon) was included in the evaluation, and the final score was determined. The patients were blinded to the scores. Figure 3 shows the application of the SCAR scale in assessing the study cases.

Considering that some patients were elderly and illiterate and may have had visual and cognitive impairments, we chose to use the NRS to assess the cosmetic appearance from the patient’s perspective. Cosmetic appearance was assessed with a numerical rating scale ranging from 0, which indicated the patient found the appearance very pleasing, to 10, which indicated the patient did not like the appearance of the scar and was not comfortable caring for the wound.

For patients who could not return to the hospital for a follow-up, according to the characteristics of the scale we selected, we used WeChat (a Chinese smartphone communication application) to obtain high-definition images and complete the corresponding assessment and help patients measure the length of their incisions.

### Statistical analysis

Statistical analysis was performed using SPSS ver. 22.0(SPSS Inc., Chicago, IL, USA). Cronbach’s alpha statistic was used to test the internal consistency of the SCAR scale and the NRS. Spearman’s rank correlation coefficient was used to estimate the inter-rater reliability of the SCAR. The convergence validity of the correlations between the SCAR scale and the NRS scores were tested using Spearman’s statistics. The baseline characteristics and scores of the SCAR scale were compared between the TA and SA groups. Variables that were normally distributed were analysed using Student’s t test, and ordinal variables and nonnormally distributed variables were analysed using the Mann-Whitney U test. All statistical tests in this study were two-sided, and P<0.05 was considered significant.

## Results

Seventy-three consecutive patients were selected for the study, 32 of whom underwent cardiac surgery with the totally thoracoscopic approach (TA group, *N* = 32) and 41 of whom underwent the median sternotomy approach (SA group, *N* = 41). All patients were followed up for at least one year. The descriptive statistics for the study population are shown in Table 2. There were no significant differences in the NYHA classification, body mass index, age, or sex between the two groups. The postoperative data are also shown in Table 2. There were no deaths among the patients included in the study. There was no significant difference in postoperative adverse events between the two groups. Three patients had bleeding events. Gastrointestinal bleeding occurred in one patient in the SA group due to anticoagulation. One patient in the TA group underwent an operation to stop the bleeding from the incision site, and another patient in the TA group experienced intercostal small vessel bleeding after drainage tube removal. One case of right femoral vein thrombosis was detected in a patient after minimally invasive surgery. The demographic characteristics and baseline clinical information were well matched between the two groups.

**Table 2:** Demographic and clinical data compared between TA group and SA group

Item	TA group	SA group	*P*
Male/Female	15/17	22/19	0.57
Age (years)	52.49 ± 10.17	51.69 ± 10.69	0.88
Current NYHA (median)	II	II	
BMI (kg/m^2^)	22.26 ± 1.51	22.49 ± 1.73	0.64
Mortality	0	0	NS
Morbidity (severe events)			
Embolism	1	0	0.44
Bleeding Events	2	1	0.58
Poor wound healing	2	2	1.00
Subcutaneous emphysema	2	0	0.18

*NYHA class* New York Heart Association functional classification, *BMI* body mass index

### Reliability and validity tests

Reliability reflects the consistency of repeated measurements. It is related to the stability and internal consistency of the measurements. In this research, Cronbach’s alpha value was used to assess internal consistency, and the Cronbach’s α value was 0.81 for the SCAR scale and 0.83 for the NRS. Cronbach’s α values greater than 0.8 indicated good agreement [13–15]. Spearman’s rank correlation coefficient was used to estimate the inter-rater reliability (Table 3). The eight subscales and the overall scores of the two groups showed strong inter-rater reliability, with statistical significance (*P* < 0.05). Convergent validity indicates the extent to which theoretically relevant scales are relevant in clinical practice [13]. Spearman’s rank coefficients were used to compare the convergent validity of the overall SCAR scores and the NRS scores. Correlation analysis revealed a significant correlation between the overall scores of the SCAR scale and the NRS scores (correlation = 0.78), and the results showed a strong positive correlation between the overall SCAR scores and the NRS scores (*P*<0.05). Patients with a lower SCAR score showed greater satisfaction with the cosmetic effects of the scar. The results showed satisfactory convergent validity.

**Table 3:** Inter-rater reliability of the SCAR scale: Spearman’s correlation analyses

SCAR parameter	Correlation coefficient	*p*
Scar spread	0.76	*P*<0.01
Erythema	0.72	*P*<0.01
Dyspigmentation	0.81	*P*<0.01
Track marks or suture marks	0.75	*P*<0.01
Hypertrophy/atrophy	0.77	*P*<0.01
Overall impression	0.82	*P*<0.01
Itch	1.00	*P*<0.01
Pain	1.00	*P*<0.01
Overall scores	0.83	*P*<0.01

Correlation coefficients: 0–0.20 = “week”; 0.21–0.40 = “fair”; 0.41–0.60 = “moderate”; 0.61–0.80 = “strong” reliability; 0.81–1.00 = “strongly” reliability [12]

### Scar assessment

The post-operative scars of cardiac surgery were evaluated using the SCAR scale and the NRS. The scores of each subscale and the lengths of the scars are shown in Table 4. There were significant differences between the two groups in the scores for the “overall impression” and “patient questions” (*P*<0.05). The “overall impression” scores were higher in the median sternotomy group than in the totally thoracoscopic group. The results showed that the scars in the totally thoracoscopic group were statistically significantly more desirable, less pain and were less itchy than those in the median sternotomy group.

**Table 4:** Cosmetic effect of surgical scars between two different approaches

parameter	TA group	SA group	*p*
Scar spread (median)	1	1	0.18
Erythema (median)	1	1	0.84
Dyspigmentation (median)	0	1	0.15
Track marks or suture marks (median)	1	1	0.31
Hypertrophy/atrophy (median)	1	1	0.54
Overall impression (median)	0	0	0.04
Patient questions (median)	0	0	0.049
Overall SCAR scores (median)	3	5	0.04
NRS scores	3.07 ± 2.40	4.80 ± 2.04	0.04
Scar length	13.42 ± 2.14	20.21 ± 2.92	P<0.01

Significant differences were found in the scores of Overall impression, Patient questions, Overall SCAR scores and NRS scores of both sides between the two groups. (*P*<0.05)

The overall scores of the SCAR scale, the scores of the NRS and the total lengths of the incisions are also listed in Table 4. There were significant differences (P<0.05) in the overall SCAR scores and the NRS scores. The overall scores of the median sternotomy group were higher than those of the totally thoracoscopic group. The mean scar length was 20.21 cm in the SA group and 13.42 cm in the TA group, with a statistically significant difference (*P*<0.05), which indicated that the length of the scar in the SA group was longer than that in the TA group.

## Discussion

Postoperative scarring can affect a patient’s health-related quality of life after surgery, and scar assessment is an integral part of assessing the cosmetic outcomes of cardiac surgery, especially in Asian populations, who are at a higher risk of developing displeasing scars [16, 17]. Studies have shown that patients often develop keloid scars at the incision site after median sternotomy [18]. Vasudev believes that large and multiple keloids are difficult to treat completely and can currently be treated with only multiple modal therapies that aim to relieve the symptoms of keloids [19]. The incidence of scar hypertrophy and scar stretch in the anterior sternal region of individuals with fair skin after open heart surgery via median sternotomy incision was studied by Elliot. The study demonstrated that scar hypertrophy and stretching often occur, and their occurrence is not related to different types of subcutaneous suture materials [20].

Generally, thoracoscopic cardiac surgery, which is commonly performed in our institution, does not damage the sternum or break the ribs, does not harm the aesthetics of the breast and is relatively invisible. Many people believe that totally thoracoscopic cardiac surgery has cosmetic advantages. However, there are few studies in relevant fields that provide detailed data. Standardized scar assessments for totally thoracoscopic cardiac surgery were conducted and evaluated in this study for the first time.

There are a number of scar scales that have been used to evaluate the condition of scars, including the Vancouver Scar Scale (VSS) [21], the Patient and Observer Scar Assessment Scale (POSAS) [22], the Manchester Scar Scale (MSS) [23], and the Stony Brook Scar Evaluation Scale [24]. Each scale has advantages and disadvantages in assessing different characteristics of scars. However, there is currently no valid and reliable scar scale to effectively assess the quality of postsurgical scars. The VSS and the POSAS were originally developed to assess burn scars and are not suitable for assessing post-surgical linear scars. Although the applicability of these scales in assessing post-surgical linear scars was later tested, the clinical considerations of these scales made at their inception were very different. Therefore, a new evaluation tool that provides a reliable outcome measure for post-surgical scars is needed. Jonathan Kantor introduced the Scar Cosmesis Assessment and Rating (SCAR) scale, which can be used to assess linear postsurgical scars in a clinical and research context. The convergent validity, inter-rater reliability and intra-rater reliability of the SCAR scale have been tested, and the results show that the SCAR scale is outstanding in terms of feasibility, validity and reliability for postoperative scar-related outcome measures [7]. The Cronbach’s alpha value of the SCAR scale in this study was 0.81. The SCAR scale and the NRS scores were convincingly reliable and valid, suggesting that the combination of the SCAR scale and NRS is a valid and reliable method for estimating scars after cardiac surgery. After the raters are briefly trained, the SCAR scale can be quickly and reliably administered during the clinical follow-up process. There is an advantage to choosing this scale; it can be assessed by photographs. A patient included in this study lived on an island and was not convenient to come to our institution, the patient uploaded photographs via a mobile phone so that the scars could be assessed [12].

Evaluations of the long-term cosmetic effects of post-operative scars are quite meaningful. Post-operative scars have a variety of final appearances, which are related to the incision site, the skin type, the suture tension, the suturing method, the wound closure technique, the surgeon’s technical ability and other factors [25]. There was no significant difference (*P* > 0.05) between the two groups of patients in our study in terms of the number of cases of poor wound healing and subcutaneous emphysema (*P* > 0.05). However, there were significant differences between the two groups in the “overall impression” and “patient question” scores. The scars in the TA group seemed more desirable, less painful and less itchy than did those in the SA group. The reason for the difference is unknown and may be related to median sternotomy, the destruction of the periosteum, the placement of a wire foreign body, additional tension caused by wire sutures to the sternum, etc. [26, 27].

**Fig. 4:**
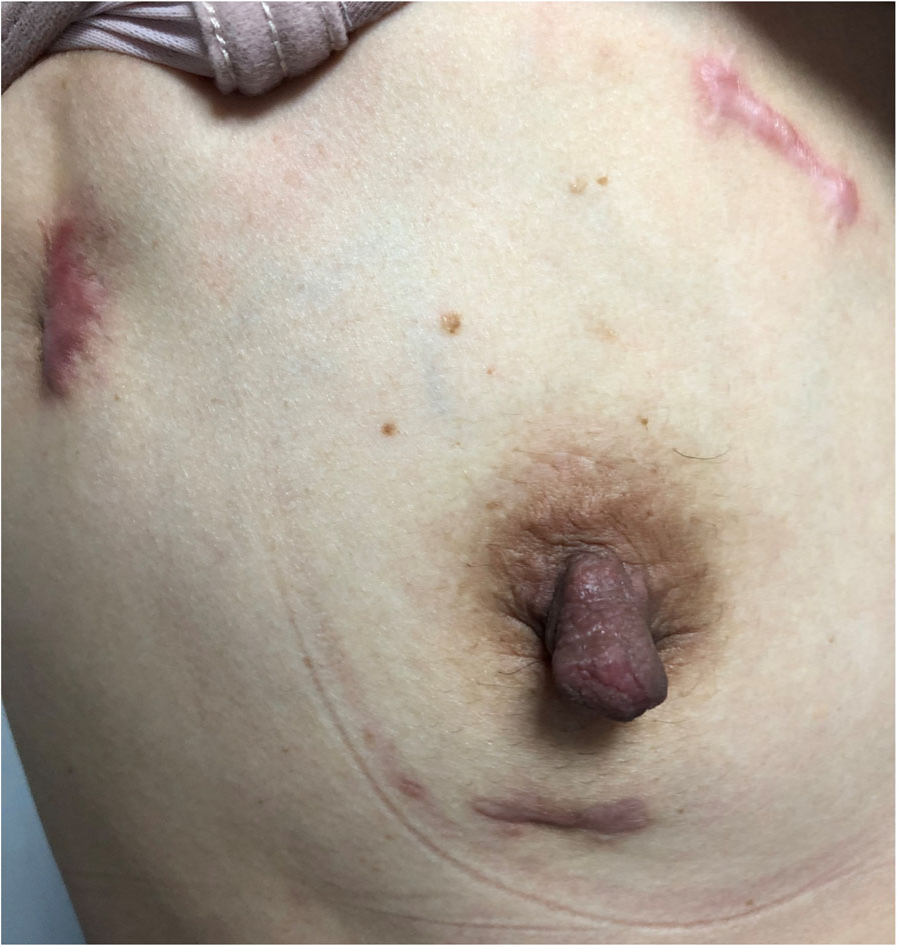
Postoperative view of reluctant chest scar hypertrophy

The average NRS score for aesthetics was quite low in both the TA and SA groups. On the other hand, the TA group had more incisions than did the SA group. The application of extracorporeal circulation and thoracoscopy in totally thoracoscopic cardiac surgery can explain the large number of incisions and their dispersion. However, the scars were apparently shorter in the TA group. In our study, we included a susceptible patient who underwent thyroid surgery and developed scar hypertrophy in the neck; she requested minimally invasive cardiac surgery, and postoperative scar hypertrophy occurred at the incision site. She was pleased with the appearance of the scar. If a median sternotomy incision was made, it was estimated that the scar hypertrophy can seriously affect the patient’s quality of life (Fig. 4).

In general, median sternotomy is the most straightforward and simplest approach, as it is easy for surgeons to perform, but totally thoracoscopic incisions are less painful and the recovery period is shorter [28]. Patients with appropriate indications can undergo cardiac surgery through the totally thoracoscopic approach, with a satisfactory scar appearance. According to the data summarized above, our study suggested that the combination of the SCAR scale and NRS is a valid and reliable tool for estimating scar appearance after cardiac surgery.

Despite these promising results, our study still has some limitations. First, some patients refused to complete the follow-ups, so selection bias may be present. Second, the cohort was small, and the follow-up period was short. In our study, cosmetic outcomes were evaluated at one year after surgery. Because the progression of scar formation is known to play a part in cosmesis and the aesthetic results continue to change over time, taking long-term follow-up pictures as often as possible is important. However, despite these limitations, our findings still provide new evidence for the selection of surgical approaches in clinical practice.

## Conclusions

The SCAR scale, in combination with the NRS, is a valid and reliable tool for estimating the cosmetic appearance of surgical scars after cardiac surgery. Scars in the TA group were considered more satisfactory and less painful and itchy than were the scars in the SA group. In addition, significant differences were found in the overall SCAR scores and the NRS scores between the two groups. Thus, according to our research, the scars of thoracoscopic surgery can lead to favourable cosmetic outcomes and patient satisfaction in the Chinese population. Patients undergoing cardiac surgery with appropriate indications who undergo the totally thoracoscopic approach can exhibit a satisfactory scar appearance.

## Data Availability

Data sharing not applicable to this article as no data sets were generated or analyzed during the current study.

## Abbreviation

SCAR: Scar Cosmesis Assessment and Rating
NRS: Numerical Rating Scale
VSS: Vancouver Scar Scale
POSAS: Patient and Observer Scar Assessment Scale
MSS: Manchester Scar Scale
NYHA class: New York Heart Association functional classification
BMI: Body mass index
